# Storm of Cardiovascular Markers After LPS Administration in Human Volunteers

**DOI:** 10.1007/s12265-021-10109-9

**Published:** 2021-03-22

**Authors:** Michael Resl, Matthias Wolfgang Heinzl, Carmen Klammer, Margot Egger, Roland Feldbauer, Johannes Pohlhammer, Benjamin Dieplinger, Martin Clodi

**Affiliations:** 1 Department of Medicine, St. John of God Hospital Linz, Seilerstaette 2, 4021 Linz, Austria; 2 Department of Laboratory Medicine, St. John of God Hospital Linz, ICMR – Institute for Cardiovascular and Metabolic Research, JKU Linz, Linz, Austria

**Keywords:** Inflammation, Infection lipopolysaccharide (LPS), Human endotoxin model, Cardiovascular, Biomarker, Proximity extension assay (PEA)

## Abstract

Acute infections are associated with an elevated cardiovascular risk. However, little is known about the interactions of acute inflammatory responses and the cardiovascular system. We therefore aimed to evaluate effects of acute inflammatory stimuli mediated by LPS administration on a set of 89 cardiovascular biomarkers. A single-blinded, placebo-controlled cross-over study using the human endotoxin model was performed. Ten healthy men were administered lipopolysaccharide (LPS) or placebo on two different study days after an overnight fast. Eighty-nine different cardiovascular biomarkers were measured repetitively over 48 h. Out of 89 cardiovascular biomarkers, 54 markers were significantly influenced by LPS infusion. The observed biomarker response to inflammation was more pronounced and complex than anticipated. In conclusion, our data show that the cardiovascular system is under enormous distress in response to experimental low-dose inflammation in humans, as demonstrated by a significant effect on 54 of the 89 biomarkers tested.

## Introduction

Atherosclerosis is a continuous inflammatory process mediated and accelerated by a variety of stimuli like lipids, diabetes mellitus, hypertension, and tobacco consumption [1]. Infiltrating inflammatory cells proliferate and secrete cytokines further triggering inflammation and the progression of fatty streaks to advanced atherosclerotic lesions. Disruption of atherosclerotic lesions exposes thrombogenic elements to the blood stream resulting in the formation of an acute or subacute thrombus, which is the most important cause for an acute cardiovascular event. Besides long acting risk factors, acute systemic inflammation plays a major role in triggering acute coronary syndromes [2]. Acute systemic infections like pneumonia have direct inflammatory effects on atherosclerotic plaques and coronary arteries [3, 4]. This has been shown in mice as well as in human studies. Acute infections promote the development of thrombi in many different ways. Systemic infections result in coronary vasoconstriction, platelet activation, and dysregulations of the coagulation system via activated protein C, plasminogen activator inhibitor type 1, and endothelial dysfunction [5].

From an epidemiological point of view, both acute respiratory infections and acute cardiovascular events vary with the seasons peaking in winter. Notably, acute respiratory symptoms precede up to a third of acute atherosclerotic events. There is a 2- to 3-fold increase in the risk of acute cardiovascular events within 1–2 weeks after a respiratory infection which might persist for up to 3 months [2, 6, 7]. In fact, this elevated risk seems to be independent of the primary cause of the systemic infection since these observations have been described in pneumonia as well as in influenza. Madjid and colleagues have shown that acute myocardial infarctions are 30% more likely to occur during influenza seasons [8]. Conversely vaccinations against influenza as well as pneumococcal infections substantially reduce the risk of acute cardiovascular events [9]. Furthermore, canakinumab, a monoclonal antibody targeting interleukin 1 beta, resulted in a significantly lower rate of recurrent cardiovascular events independent of lipid lowering therapy [10].

In summary, it appears to be quite clear that acute infections and inflammatory conditions present an important cardiovascular risk factor or may even exert a direct effect causing cardiovascular events. However, most pathophysiological pathways and molecular triggers of this epidemiologically proven association between systemic infections and acute cardiovascular events as well as diagnostic markers to predict such risk under inflammatory circumstances remain to be elucidated.

We thus aimed to evaluate the impact of an acute inflammatory stimulus mediated by lipopolysaccharide (LPS) infusion on cardiovascular biomarkers in healthy human volunteers.

## Methods

The study was approved by the local Institutional Review Board (research ethics committee of the St. John of God Hospital Linz) as well as the ethics committee of the Medical University of Vienna. Informed consent was obtained orally and in writing from each subject before enrolment in the study.

## Protocol

The study was performed as a single-blinded, prospective, placebo-controlled, randomized, cross-over study. Ten healthy non-smoking male probands (aged 18 to 40 years) were included. In a random order and single-blinded manner, subjects were administered intravenous bacterial endotoxin (2 ng/kg National Reference Bacterial Endotoxin [LPS]) and intravenous placebo (saline 0.9%) on two different study days, separated by a washout phase of at least 14 days. The study was performed at 08.00 h after an overnight fast. The volunteers were allowed to drink non-sparkling mineral water during the study day and were allowed to eat after the respective study day (6 h after infusion). Continuous monitoring (electrocardiogram, heart rate, temperature, and blood pressure) was performed while subjects remained in a supine position.

US Standard Reference Endotoxin (lot #94332B1) was kindly provided by the Investigational Drug Management at the National Institutes of Health (NIH), Bethesda, Maryland. Endotoxin was shipped in vials as a white, sterile, lyophilized powder and was reconstituted at our institution according to the recommendations of the manufacturer.

### Blood Sampling, Laboratory Measurements, and Statistical Analysis

Blood samples for the measurement of cardiovascular biomarkers were taken repetitively after infusion at 0, 15, 30, 45, 60, 90, 120, 180, 240, and 360 min as well as 24 h and 48 h after infusion. The last two samples of blood were taken at 08:00 h in the morning after overnight fasting.

Using VACUETTE polyethylene terephthalate glycol blood collection tubes (Greiner Bio-One), EDTA plasma was collected and subsequently stored at −80 °C.

For the analysis of the cardiovascular biomarker panel, aliquots were sent to Olink proteomics (Upsala, Sweden), where analysis of the cardiovascular biomarker panel was performed using the proximity extension assay (PEA) technology.

PEA is an immunoassay based on pairs of oligonucleotide-labeled antibodies (“probes”). As the antibodies bind to their specific antigens, the oligonucleotides hybridize. A new PCR target sequence is thus formed by a proximity-dependent DNA polymerization, and quantification is performed by quantitative real-time PCR (qPCR). Biomarker concentrations are given in NPX units (normalized protein expression), an arbitrary unit on a log2-scale. Thus, an increase by one NPX unit reflects a doubling of biomarker concentration.

Statistical analysis was performed using IBM SPSS Statistics 25. Statistical tests included paired *t* tests and repeated-measures analysis of variance (RM-ANOVA). Benjamini–Hochberg correction was applied to correct for multiple testing.

## Results

### Proband Characteristics

All probands were recruited from December 2017 until June 2018. Altogether, 24 volunteers were screened, of which 6 were excluded after screening and 8 chose not to participate. The mean age was 24.1 (SD 3.7) years, and the average body mass index (kg/m^2^) was 25.2 (SD 1.6).

### Inflammation and Validation After LPS Infusion

Both study days were completed successfully in all 10 subjects who chose to participate after the screening examination. As previously described, induction of experimental inflammation was successful in all 10 volunteers, as reflected by a significant increase of IL-6 as a rapid marker of inflammation, with a peak at 180 min after LPS infusion.

### Cardiovascular Biomarkers

**Fig. 1:**
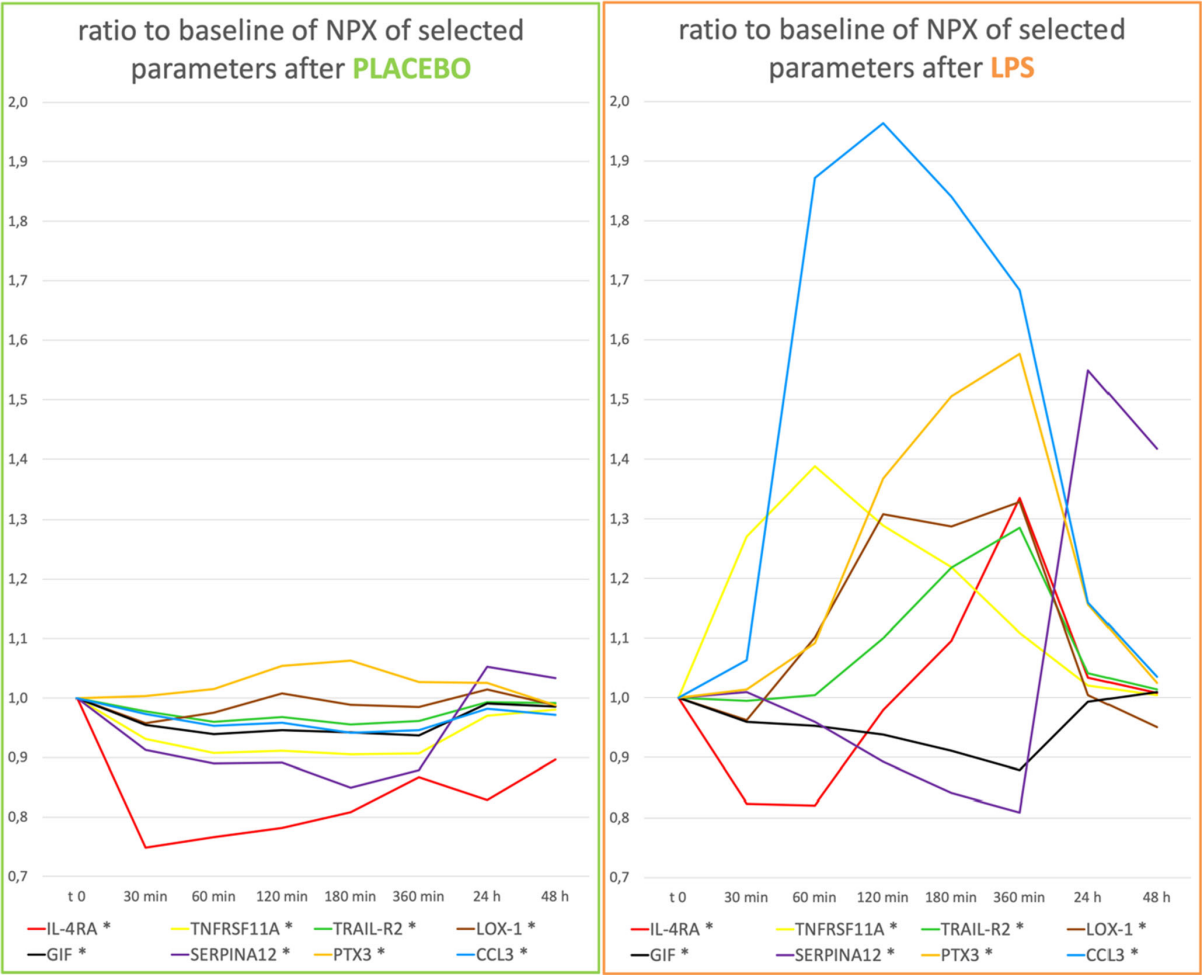
Cardiovascular biomarkers following placebo or LPS infusion. All parameters marked with “*” show significant differences after LPS infusion in comparison to placebo. Biomarkers were measured in NPX values (normalized protein expression), which is an arbitrary unit on a log-2 scale. Of note, values on the *y*-axis do not depict the NPX values but ratio-to-baseline in order to show the relative changes in a normalized manner. This means that changes on the logarithmic NPX scale are much larger than they appear in this graph. Parameters marked with “*” are significantly influenced by the administration of LPS in comparison to placebo

The measurement of 89 cardiovascular biomarkers showed a significant change in 54 markers following LPS administration as compared with placebo (Fig. 1).

All markers presented below result in a *p*-value of *p* < 0.05 after Benjamini–Hochberg correction.

#### Markers of angiogenesis and blood vessel morphogenesis

Growth differentiation factor 2 (GDF-2), vascular endothelial growth factor D (VEGFD), heme oxygenase 1 (HO-1), decorin (DCN), placenta growth factor (PGF), adrenomedullin (ADM), tissue factor (TF)

#### Markers of catabolic processes

Matrix metalloproteinase-7 (MMP-7), alpha-l-iduronidase (IDUA), decorin (DCN), protein AMBP (AMBP), heme oxygenase 1 (HO-1), cathepsin L1 (CTSL1), brother of CDO (BOC), receptor for advanced glycosylation end products (RAGE), a disintegrin and metalloproteinase with thrombospondin motifs 13 (ADAM-TS13), angiotensin-converting enzyme 2 (ACE2), matrix metalloproteinase-12 (MMP-12), lipoprotein lipase (LPL), prolargin (PRELP)

#### Markers of cell adhesion

Receptor for advance glycosylation end products (RAGE), T-cell surface glycoprotein CD4 (CD4), galectin 9 (Gal-9), lymphotactin 1 (XCL1), SLAM family member 7 (SLAMF7), programmed cell death 1 ligand 2 (PD-L2), Interleukin 27 (IL-27), P-selectin glycoprotein ligand 1 (PSGL-1), interleukin-1 receptor antagonist protein (IL-1ra), stem cell factor (SCF), ADAM-TS13, lectin-like oxidized LDL receptor 1 (LOX-1), interleukin-4 receptor subunit alpha (IL-4RA), interleukin-1 receptor-like 2 (IL1RL2), spondin-2 (SPON2), BOC

#### Markers of coagulation

Thrombomodulin (TM), ADAM-TS13

#### Markers of heart development

ADM

#### Markers of immune response

Tumor necrosis factor receptor superfamily member 13B (TNFRSF13B), IL1RL2, low affinity immunoglobulin gamma Fc region receptor II-b (IgG Fc receptor II-b), C–C motif chemokine 3 (CCL3), carcinoembryonic antigen related cell adhesion molecule 8 (CEACAM8), GAL-9, HO-1, Il-4RA, SLAMF7, tumor necrosis factor receptor superfamily member 10A (TNFRSF10A), IL-27, tumor necrosis factor receptor superfamily member 11A (TNFRSF11A), AMBP, SPON2, macrophage receptor MARCO (MARCO), ADM, C–C motif chemokine 17 (CCL17), C–X–C motif chemokine 1 (CXCL1), ADAM-TS13, RAGE, PD-L2, osteoclast-associated immunoglobulin-like receptor (hOSCAR), pentaxin-related protein PTX3 (PTX3), pro-interleukin-16 (IL16), TNF-related apoptosis-inducing ligand receptor 2 (TRAIL-R2)

#### Markers of inflammatory response

XCL1, CXCL1, IL1RL2, LPL, PTX3, TRAIL-R2, RAGE, LOX-1, CCL3, TNFRSF11A, IL27, TNFRSF10A, GAL-9, interleukin-17D (IL-17D), TNFRSF10A, Gal-9, C–C motif chemokine 17 (CCL17), IL-1ra, HO-1, ACE2

#### Markers of MAPK cascade

SCF, CCL3, renin (REN), TNFRSF11A, Gal-9, XCL1, CCL17, AMBP

#### Markers of platelet activation

TM, ADAM-TS13

#### Markers of proteolysis

ADAM-TS13, TF, LOX-1, MMP12, chymotrypsin (CTRC), TNFRSFA10A, REN, TRAIL-R2, MMP7, ACE2

#### Markers of regulation of blood pressure

ACE2, REN, HO-1

#### Markers of response to hypoxia

VEGFD, ADM, HO-1, PGF

#### Markers of response to peptide hormones

Serpin A12 (SERPINA12), agouti-related protein (AGRP), sortilin (SORT1), ADM

#### Markers of wound healing

TM, TF, MMP12, ADAM-TS13, HO-1, DCN

#### Other markers

Follistatin (FS), gastric intrinsic factor (GIF), pappalysin-1 (PAPPA), V-set, and immunoglobulin domain-containing protein 2 (VSIG2)

## Discussion

Fifty-four out of 89 cardiovascular biomarkers showed a highly significant change in an LPS mediated model of inflammation in healthy volunteers.

**Fig. 2:**
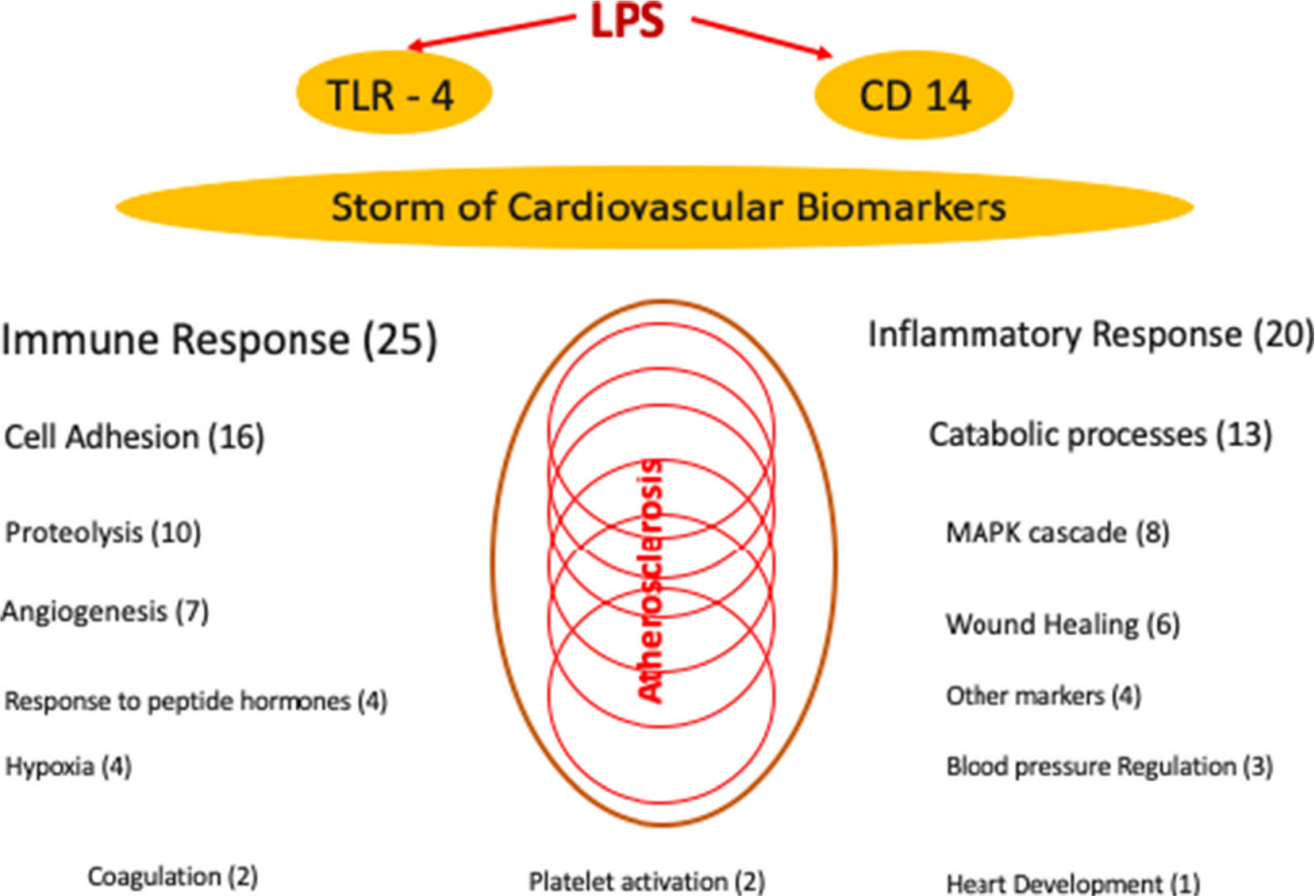
Significant effects of LPS on cardiovascular biomarkers, showing number of affected markers per group

Many different categories of cardiovascular biomarkers like markers of angiogenesis, catabolic processes, cell adhesion, coagulation system, immune response, and even markers of hypoxemia are affected. The fact that LPS has such a tremendous influence on most of the currently known cardiovascular biomarkers is difficult to interpret. Since there is a dynamic interaction of the proteins measured, we cannot establish direct relations of different pathophysiologic pathways (Fig. 2). The actual understanding of the LPS—TLR4-NFkB—pathway just partially explains our findings. It seems that there are many currently unknown pathways being responsible for the interplay of inflammatory stimuli and the cardiovascular system.

However, our data underscore the findings of important clinical studies showing that bacterial as well as viral infections tremendously increase the risk of cardiovascular events [2, 3]. In influenza patients, admissions for acute myocardial infarction were six times as high during the 7 days after laboratory confirmation of influenza infections (20 admissions per week vs. 3.3 admissions per week) [11]. Conversely, it has been shown that influenza vaccinations reduce cardiovascular mortality (RR 0.45; 95% confidence interval 0.26–0.76 *p* = 0.003) [9].

Among patients being hospitalized for pneumococcal pneumonia, Musher and colleagues found a rate of myocardial infarction of 7 to 8% [3]. This association seems to be independent of the cause of pneumonia and has also been established for Haemophilus influenza pneumonia and in patients with pneumonia from any cause [4, 12, 13]. After pneumonia, the cardiovascular risk decreases within the first months but still exceeds the baseline risk up to 10 years after infection. Autopsy studies and animal studies have shown that inflammatory activity increases in atherosclerotic plaques after infections [5, 8]. Systemic inflammation promotes an oxidative burst contributing to plaque destabilization [14].

It seems that acute as well as chronic inflammatory processes have a more significant influence on the cardiovascular system than expected, as shown by epidemiologic data and the results of our study. Observational studies have also shown that concomitant therapy with glucocorticoids and blockers of the renin angiotensin system reduced the risk of post-pneumonic myocardial infarction [15, 16]. Evaluation of the biomarker panel in patients suffering from infections might select high-risk patients especially benefitting from an intensified cardiovascular risk factor therapy after an infection.

Since their development, cardiovascular biomarker panels have broadly been applied in clinical studies. In the ORIGIN study, a panel of 237 cardiovascular biomarkers was analyzed showing that, finally, 10 out of 237 markers were significant predictors of cardiovascular outcomes [17].

In comparison to the results of the ORIGIN study administration of LPS, a model of inflammation significantly increased 54 out of 89 biomarkers speaking for the pronounced effect of inflammation on the cardiovascular system.

The interplay between inflammation and cardiovascular risk seems to be of particular interest at the moment with regard to the current COVID-19 pandemic causing highly elevated mortality rates worldwide.

In this study, we show effects of inflammation on cardiovascular parameters. However, the interplay between inflammation and the cardiovascular system may also exist vice versa, considering that patients with preexisting cardiovascular disease are at greater risk from COVID-19 with higher case-fatality rates and more severe disease [18].

Interestingly, 24 h after administration of LPS, the storm of cardiovascular biomarkers has already disappeared. This, of course, is a considerable limitation of our study since bacterial and viral infections usually last longer than just 24 hours.

However, it is only in an experimental setting like the human endotoxin model applied in our study that effects of specific stimuli can be studied.

To the best of our knowledge, none of the previously conducted studies has evaluated such a large set of different biomarkers in relation to LPS administration [19, 20].

In summary, interactions of inflammation and the cardiovascular system are much more dynamic and complex than expected, speaking for the poor understanding of the epidemiologically shown association of systemic infections and cardiovascular events.

Future cardiovascular biomarker panel studies in patients suffering from infections might select a high-risk collective of patients who may possibly benefit from cardioprotective therapies like statins or immune modulatory therapies during and following serious infections.
